# Comparison of Key Properties of Ag-TiO_2_ and Hydroxyapatite-Ag-TiO_2_ Coatings on NiTi SMA

**DOI:** 10.3390/jfb15090264

**Published:** 2024-09-12

**Authors:** Karolina Dudek, Aleksandra Strach, Daniel Wasilkowski, Bożena Łosiewicz, Julian Kubisztal, Anna Mrozek-Wilczkiewicz, Patryk Zioła, Adrian Barylski

**Affiliations:** 1 Łukasiewicz Research Network-Institute of Ceramics and Building Materials, Cementowa 8, 31-983 Kraków, Poland; 2 Doctoral School, University of Silesia, Bankowa 14, 40-032 Katowice, Poland; aleksandra.strach@us.edu.pl; 3 Institute of Biology, Biotechnology and Environmental Protection, Faculty of Natural Sciences, University of Silesia, Jagiellońska 28, 40-032 Katowice, Poland; daniel.wasilkowski@us.edu.pl; 4 Institute of Materials Engineering, University of Silesia, 75 Pułku Piechoty 1A, 41-500 Chorzów, Poland; bozena.losiewicz@us.edu.pl (B.Ł.); julian.kubisztal@us.edu.pl (J.K.); adrian.barylski@us.edu.pl (A.B.); 5 Department of Systems Biology and Engineering, Silesian University of Technology, Boleslawa Krzywoustego 8, 44-100 Gliwice, Poland; anna.mrozek-wilczkiewicz@us.edu.pl; 6 August Chełkowski Institute of Physics, University of Silesia, 75 Pułku Piechoty 1, 41-500 Chorzów, Poland; patryk.ziola@us.edu.pl

**Keywords:** Ag-TiO_2_ coating, hydroxyapatite, wettability, adhesion, surface nanoroughness, antimicrobial studies, cell viability, corrosion resistance, electronic properties

## Abstract

To functionalize the NiTi alloy, multifunctional innovative nanocoatings of Ag-TiO_2_ and Ag-TiO_2_ doped with hydroxyapatite were engineered on its surface. The coatings were thoroughly characterized, focusing on surface topography and key functional properties, including adhesion, surface wettability, biocompatibility, antibacterial activity, and corrosion resistance. The electrochemical corrosion kinetics in a simulated body fluid and the mechanisms were analyzed. The coatings exhibited hydrophilic properties and were biocompatible with fibroblast and osteoblast cells while also demonstrating antibacterial activity against *E. coli* and *S. epidermidis*. The coatings adhered strongly to the NiTi substrate, with superior adhesion observed in the hydroxyapatite-doped layers. Conversely, the Ag-TiO_2_ layers showed enhanced corrosion resistance.

## 1. Introduction

NiTi alloys have gained wide recognition in the medical field due to their unique mechanical properties, such as the shape memory effect and superelasticity. These mechanical properties make them exceptionally well-suited for orthopedic implants, more than other metals [1]. However, one of the main challenges associated with the use of NiTi shape memory alloys (SMA) in medicine is their potential cytotoxicity due to the release of nickel ions, which can cause allergic and inflammatory reactions. Additionally, the NiTi surface may be susceptible to corrosion in the aggressive environment of body fluids, leading to material degradation. Therefore, surface modification of NiTi alloys to enhance their biocompatibility has become a crucial area of research [2].

In recent years, techniques for producing multifunctional layers on the surfaces of various titanium alloys have been developing intensively [3]. These modifications aim to impart additional properties to the surface, such as antibacterial activity, bioactivity, or the controlled release of drugs. Composite coatings, which combine different materials with distinct properties, show the greatest potential. For coatings applied to NiTi shape memory alloys, it is crucial that they remain thin and flexible to preserve these unique properties [4]. Consequently, there is an increasing focus on surface modifications using nanomaterials.

Hydroxyapatite and titanium oxides are widely used to modify the surfaces of orthopedic implants. Hydroxyapatite enhances biocompatibility and accelerates osseointegration. Hydroxyapatite coatings facilitate the proliferation and differentiation of osteogenic cells on the implant surface, thereby augmenting its stability and long-term integration with bone tissue [5,6]. Nano hydroxyapatites provide higher surface area and reactivity in implementation [7]. Furthermore, hydroxyapatite nanoparticles are being extensively researched as a carrier material for controlled drug delivery systems. Their application allows for the localized administration of therapeutic agents, including antibiotics, growth factors, and other pharmaceuticals, directly at the implantation site, thereby optimizing therapeutic efficacy and minimizing systemic side effects [8,9]. Nanostructured titanium oxide coatings can also significantly increase the contact area between the implant and the surrounding tissues, thereby enhancing integration and stability [10,11]. Additionally, modified nanometric titanium oxides, such as those incorporating various phases containing silver, can impart antibacterial properties, reducing the risk of infection around the implants [12,13,14].

When designing new biomaterials, numerous critical factors must be considered to ensure their efficacy and safety in medical applications. The composition (both chemical and phase), quality, morphology, and surface topography (especially the roughness) play pivotal roles in determining how well the implant is accepted by the body and influences cellular metabolism processes.

The roughness of the coatings is a parameter that significantly impacts osseointegration [15,16,17]. Optimal roughness enhances the adhesion of bone cells to the implant surface, thereby promoting its stability and functionality. Surfaces that are too smooth may not provide adequate cell adhesion, while excessively rough surfaces may create micro-spaces that can harbor bacteria [6,18,19]. Surface nanoscale roughness, which is comparable in scale to proteins and cell-membrane receptors, plays a crucial role in osteoblast differentiation and tissue regeneration. This nanoscale roughness can significantly influence cellular behaviors, such as adhesion, proliferation, and spreading [20,21,22,23].

Additionally, surface wettability is crucial in shaping implant properties. Surface wettability significantly influences the adhesion of proteins and other macromolecules to the surface, known as conditioning. It also affects the interactions between hard and soft tissue cells and the implant surface, bacterial adhesion and subsequent biofilm formation, and the rate of osseointegration [24,25,26,27]. Hydrophilic surfaces generally promote the early stages of cell adhesion, proliferation, differentiation, and bone mineralization more effectively than hydrophobic surfaces [28].

Surface wettability is crucial for bacterial adhesion. The adhesion of human pathogens, such as *S. aureus*, *S. epidermidis*, and *E. coli*, correlates with increased surface hydrophobicity [29,30]. Therefore, it is desirable for biomaterial surfaces to be hydrophilic. Moreover, it is desirable for implants to possess antibacterial properties. This can be achieved through the gradual release of antibacterial agents, drugs, or elements, such as silver [12,13,14].

To ensure implant acceptance by the human body and support the metabolic processes in surrounding tissues, it is crucial to assess the biocompatibility of an implant’s surface. It is also crucial to verify whether the modification promotes the desired cell adhesion and influences cell morphology. Proper adhesion is fundamental for most normal body cells, supporting processes such as cell movement and intercellular communication. Additionally, it is vital for cell survival and physiological function. Adhesive proteins play a significant role in regulating cellular processes, including receptor recognition, immune responses (e.g., during inflammation), and apoptosis. Consequently, any malfunction of these proteins can lead to disturbances in cellular function, ultimately affecting the biological response of the human body [31]. In addition, cell adhesion is closely related to surface wettability and roughness [20,21,22].

Furthermore, assessing mechanical properties and, for coated implants, ensuring strong adhesion between the coating and the metallic substrate are important. Good adhesion is essential for guaranteeing the durability and functionality of the implant.

Corrosion is a significant challenge to the use of metals and metal alloys for various types of implants. The corrosion of implants in the environment of body fluids is primarily due to their aggressive nature. Body fluids contain ions such as chlorine, sodium, potassium, calcium, and magnesium, as well as phosphates. The aggressiveness of this biological milieu is intensified by the presence of organic ingredients, such as proteins. Additionally, implants must function at the constant, relatively high temperature of the body and endure various loads and tribological conditions [3]. Under normal conditions, the body’s pH is approximately 7.4, but the introduction of a foreign body can cause the pH at the implant site to become acidic. This combination of factors creates a highly demanding environment that not all materials can withstand. Corrosion products may be secreted or retained in the body, accumulating in tissues surrounding the implant and causing local tissue reactions, or they may move passively through tissues and the circulatory system, potentially being actively carried by macrophages and accumulating away from the implant site [32].

In this work, the study conducted a comprehensive comparative characterization of innovative layers deposited on NiTi alloy through electrophoresis. The first coating consisted of Ag-TiO₂ nanoparticles [33], while the second layer incorporated Ag-TiO₂ with hydroxyapatite, featuring nanometric and submicrometer particle sizes [34]. The coatings were extensively analyzed for their functional properties, including adhesion, topography, wettability, biocompatibility, and antibacterial efficacy. Furthermore, their corrosion resistance properties were extensively discussed.

## 2. Materials and Methods

### 2.1. Materials

The research material used consisted of Ag-TiO_2_ and hydroxyapatite-Ag-TiO_2_ layers produced by electrophoretic deposition (EPD) on the passivated surface of a NiTi shape memory alloy. Ag-TiO_2_ coatings were produced by anaphoresis at a voltage of 40 V for 3 min, while HAp-Ag-TiO_2_ layers were produced by cataphoresis at a voltage of 20 V for 1 min. The deposited coatings were heat-treated at 800 °C for 2 h. A detailed description of the production of Ag-TiO_2_ layers is provided in publication [33] and of HAp-Ag-TiO_2_ layers in publication [34].

### 2.2. Methods of Testing

#### 2.2.1. Adhesion

The scratch resistance of the coatings was tested using the Micro Combi Tester—MCT3 device from Anton-Paar (Corcelles-Cormondrèche, Switzerland) using the scratch-test method. Test procedures were performed in accordance with the guidelines of [35,36,37,38] ISO 19252:2008, ISO 20502:2005, ASTM C1624-22(2022), and ASTM D7027-20(2020), using a Rockwell diamond indenter with a diameter of 100 μm. Each test was divided into three stages. In the first stage (pre-scan), the sample profile was scanned under a load of 0.03 N. In the second stage (scan), proper tests were carried out, with a progressively increasing load from 0.03 to 30 N. The length of each scratch was 6 mm, and the speed indenter movement was 12 mm/min. The last stage (post-scan) consisted of scanning the profile under a load of 0.03 N, resulting from scratching the surface. During the tests, the following parameters were recorded: friction force—F_t_ [N] and acoustic emission—AE [%]. Average values were derived from 3 measurements. The surface after the tests was imaged using a scanning microscope.

Scanning electron microscopy (SEM) data obtained by a TESCAN Mira 3 LMU were used to determine the microstructure of the coatings after the scratch tests. Images were collected by secondary electrons (SE) and backscattered electrons (BSE). The measurements were carried out on the samples covered by a 5 nm Cr layer by using Quorum Q150T ES equipment.

#### 2.2.2. Topography

The coating surfaces were imaged using the Park NX7 atomic force microscope (AFM) from Park Systems Corporation (Suwon, Republic of Korea). The sample surface was scanned in Tapping mode utilizing an NSC18 probe (spring constant k = 5 N/m, resonance frequency 160 kHz). Images were acquired from areas of 10 µm × 10 µm, 3 µm × 3 µm, and 750 nm × 750 nm. The roughness parameters were then calculated based on these images.

#### 2.2.3. Wettability

Contact angle (CA) measurements were performed using an OCA 15EC goniometer with an accuracy of ±0.01° using the sessile drop method. Ten images of a water drop with a volume of 1 µL placed on the tested surface were recorded over 5 s. Based on these images, the average contact angle (CA) values were calculated. The final contact angle values were taken as the average of ten measurements made in different areas of the tested surface.

#### 2.2.4. Biocompatibility

To perform biocompatibility, tests of the normal human dermal fibroblast (NHDF) and the human osteoblasts (HOB), obtained from PromoCell (Heidelberg, Germany) were used. The cells were grown as monolayer cultures in 75 cm^2^ flasks (Nunc, Roskilde, Denmark). NHDF were cultured in Dulbecco’s Modified Eagle’s Medium (Sigma, Oakville, ON, Canada) supplemented with 15% of the non-inactivated fetal bovine serum (Sigma, Oakville, ON, Canada), while HOBs were grown in the complete osteoblast medium (Sigma, Oakville, ON, Canada). Both mediums contained a standard set of antibiotics (1% *v*/*v* of penicillin and streptomycin) (Gibco, Carlsbad, CA, USA). The cells were grown under standard conditions at 37 °C in a humidified atmosphere at 5% of CO_2_.

To perform cytotoxicity of the coatings, the tested samples in the form of coated disks were placed in a 24-well plate (Nunc, Roskilde, Denmark). The cells were then seeded at a density of 25,000 cells/well and incubated at standard growing conditions for 48 h. The reference disks (without coating) served as a reference surface. After 48 h, the culture medium was replaced with DMEM medium without phenol red, and CellTiter 96^®^AQueous One Solution-MTS (Promega, Gutenbergring, Germany) solution was added to each well. One hour later, the absorbance of that synthesized in the living cells’ formazan was measured at 490 nm using a multi-plate reader Varioskan LUX (Thermo Fisher Scientific, Waltham, MA, USA). The obtained results are expressed as a percentage of the reference material—an uncoated disk. Each material polymer was tested in triplicate in a single experiment with each experiment being repeated three times.

The NHDF and HOB cell lines were seeded on the tested disks as described above. After 48 h, the culture medium was replaced by a mixture of 1 μM dye, Calcein-AM, and 4 μg/mL Hoechst 33342 (both from Thermo Fisher Scientific, Waltham, MA, USA) prepared in DMEM without phenol red. After 15 min of incubation, the samples with attached cells were washed three times with the PBS, and then, the materials were transferred to the optic slide. Visualization of cells using a 488 nm excitation laser, a 520 nm emission filter, a 350 nm excitation laser, and a 461 nm emission filter was carried out using a Zeiss AxioObserver Z1 inverted fluorescence microscope equipped with an AxioCam RMn camera (Carl Zeiss AG, Jena, Germany).

#### 2.2.5. Microbiological Investigations

**Figure 1:**
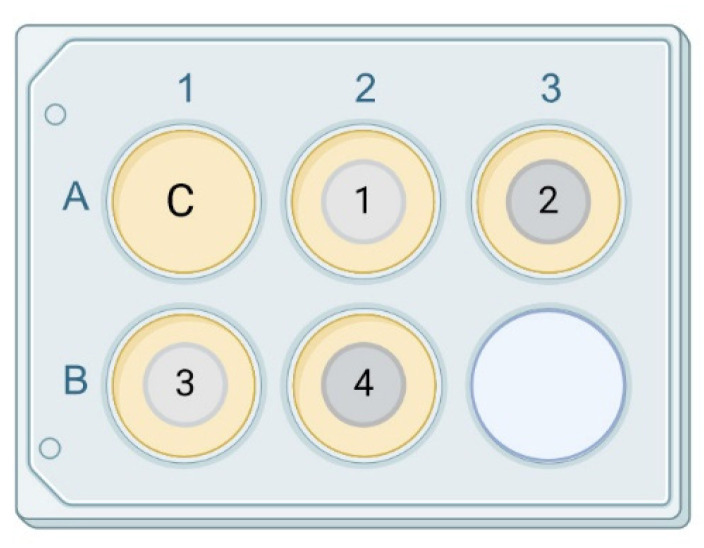
Schematic presentation of experiments, including non-exposed bacterial culture (control) (C), NiTi substrate with *E. coli* (1), HAp coating with *E. coli* (2), Ag-TiO_2_ coating with *E. coli* (3), and HAp-Ag-TiO_2_ coating with *E. coli* (4). Created in biorinder.com.

To perform the microbiological investigation, two bacterial strains were tested for responses to the exposure of selected materials. They included Gram-negative *Escherichia coli* (ATCC 25922) and Gram-positive *Staphylococcus epidermidis* (ATCC 12228) strains purchased from the American Type Culture Collection (ATCC). All these strains were exposed to four types of materials, including NiTi substrate (1), HAp coatings (2), Ag-TiO_2_ coatings (3), and HAp-Ag-TiO_2_ coatings (4). The assays were performed under sterile conditions. The tested materials were placed into separate wells of a 6-well plate, to which a bacterial suspension of OD_600_ = 0.1 (~107 CFU mL^−1^) was added, fully immersing the test material (Figure 1). The bacterial systems and tested materials were incubated for 24 h at 37 °C and continuous shaking at 140 rpm.

The presented studies were performed in two ways. One approach was to mimic the non-favorable conditions present in the organism using Ringer’s solution (sodium chloride 8.6 g L^−1^, calcium chloride 0.3 g L^−1^, and calcium chloride dihydric 0.33 g L^−1^) during a 24 h incubation. The second approach was using a dedicated culture media during a 24 h incubation of the bacteria with the analyzed material. For *E. coli*, the media used was Bacto™ Tryptic Soy Broth (cat. no. 211825; cat. 211825; pancreatic digest of casein 17.0 g L^−1^, papaic digest of soybean 3.0 g L^−1^, dextrose 2.5 g L^−1^, sodium chloride 5.0 g L^−1^, and dipotassium phosphate 2.5 g L^−1^) and *S. epidermidis* substrates Difco™ Nutrient Broth (Cat. No. 234000; beef extract 3.0 g L^−1^, peptone 5.0 g L^−1^). In the next step, a certain amount of suspension was taken after 24 h of incubation, and cultures were performed on media dedicated to the specific bacterial strain, including *E. coli* with Bacto™ Tryptic Soy Broth and *S. epidermidis* with Difco™ Nutrient Broth. Afterward, bacterial colonies were counted, and the number of bacteria was expressed as colony-forming units-CFU mL^−1^. The statistical significance analysis used a Student’s *t*-test for independent samples (Statistica 13.3 TIBCO).

#### 2.2.6. The Corrosion Resistance

The in vitro corrosion resistance of the investigated electrodes was tested in a simulated body fluid known as Ringer’s solution, which contained specific amounts of sodium chloride—8.60 g L^−1^, potassium chloride—0.30 g L^−1^, and calcium chloride dihydrate—0.33 g L^−1^ [39]. The solution’s pH was adjusted to a physiological level of 7.4 ± 1.0 using a 4% sodium hydroxide solution and a 1% lactic acid solution, in accordance with international standard [40] ISO 10271:2021. Analytical grade chemicals (Avantor Performance Materials Poland S.A., Gliwice, Poland) and ultrapure water with a conductivity of 0.055 μS cm^−1^ at 25 °C generated by the Milli-Q Advantage A10 Water Purification System (Millipore SAS, Molsheim, France) were used to prepare the solution. The electrochemical measurements were conducted at 37.0 ± 0.1 °C using a thermostat with the Ringer’s solution being purged with argon of 5.0 purity for 30 min to remove dissolved oxygen.

Corrosion-resistance measurements were conducted using a three-electrode configuration, with the electrode being tested as the working electrode (WE), a platinum foil (8 cm^2^) as the counter electrode (CE), and a saturated calomel electrode (SCE) as the reference electrode (RE). The RE was introduced into the Ringer’s solution through a Luggin capillary. The geometric surface area of the WE was 1.5 cm^2^. The method of preparing the electrodes and their positioning within the cell was provided in the previous study [32]. An argon flow was maintained above the surface of Ringer’s solution to minimize the reintroduction of air into the electrochemical cell.

All electrochemical measurements were performed with a modular high-current Potentiostat–Galvanostat Electrochemical System with a maximum current of 1 A and a compliance voltage of 30 V (Metrohm Autolab B.V., Utrecht, The Netherlands). The open circuit potential (E_OC_) was stabilized for 1 h. The E_OC_ was treated in further studies as the approximate corrosion potential (E_cor_).

Electrochemical impedance spectroscopy (EIS) measurements were conducted using FRA-coupled potentiostat at the E_OC_ in a wide range of frequency (f) covering 50 kHz–1 mHz, with 10 frequencies per decade at a resolution of f equal to 0.003%. A small voltage perturbation applied to the system was 5 mV. The physico-chemical modeling of the experimental EIS characteristics was performed using the concept of equivalent electrical circuits. The data obtained from this alternating current (AC) method were fitted using the complex non-linear least squares (CNLS) method with modulus weighting [41]. The CNLS-fitting was carried out using the EQUIVCRT program, with the significance of the fitting elements checked using the F test.

CNLS modeling is a technique used in EIS studies to analyze and interpret impedance data [41]. It involves fitting the data to an equivalent electrical circuit model using a nonlinear least squares algorithm. This process helps in extracting meaningful information from the complex impedance data. The key features of CNLS modeling include:

1. Equivalent circuit models:
  (a) Basics: the models represent the electrochemical behavior of the system using components like resistors (R), capacitors (C), inductors (L), and constant phase elements (CPE);
  (b) Complexity: the complexity of the model can vary depending on the system, with simple systems requiring fewer components and more complex systems needing a larger number of elements.
2. Nonlinear least squares fitting:
  (a) Objective: to minimize the difference between the experimental and modeled impedance data;
  (b) Algorithm: this involves iteratively refining the parameters of the equivalent circuit using a nonlinear least squares algorithm to reduce the sum of squared differences.
3. Complex impedance:
  (a) Real and imaginary parts: CNLS modeling considers both the resistive and reactive components of impedance;
  (b) Phase angle: the phase angle between the applied current and resulting potential is also taken into account for a complete description of the system’s impedance.
4. Advantages:
  (a) Accuracy: CNLS modeling provides a precise fit to the experimental data;
  (b) Insight: the fitted parameters offer insights into the system’s electrochemical processes;
  (c) Versatility: CNLS modeling can be applied to a wide range of electrochemical systems.
5. Software Tools:
  (a) Commercial software: packages like ZView, EC-Lab, and Gamry Echem Analyst incorporate CNLS modeling;
  (b) Custom scripts: researchers can develop custom scripts using languages like Python, MATLAB, or R for greater flexibility.

The steps in CNLS modeling include data acquisition, preliminary analysis, model selection, CNLS fitting, parameter extraction, and validation of the model by comparing the fitted data with the experimental data.

In direct-current (DC) measurements using the cyclic potentiodynamic polarization method, the potential was scanned at a polarization rate of v = 1 mV s^−1^, starting from a potential 200 mV more negative than the E_OC_ to the breakdown potential (E_bd_), at which pitting corrosion initiated on the electrode surface. Once the E_bd_ was reached, the direction of polarization was reversed towards more negative values. This reverse scan was continued until the protection potential (E_p_) was reached, at which the pitting stopped and the electrode surface was considered to be repassivated or protected from further pitting corrosion. The resulting current–potential curves in a semi-logarithmic system were analyzed to determine key corrosion-resistance parameters after smoothing by the Savitzky–Golay algorithm using the General Purpose Electrochemical System (GPES) for Windows—Version 4.9 software [42].

#### 2.2.7. The Electronic Properties

The electronic properties of the tested materials were evaluated using the scanning Kelvin probe (SKP) technique in an air environment using a PAR Model 370 Scanning Electrochemical Workstation (Prinston Applied Research, Oak Ridge, TN, USA), which has an integrated SKP370 module and a VCAM3 optical video microscope. A U-SKP-150 tungsten microprobe (Uniscan Instruments, Buxton, UK), featuring a brass housing and a tungsten wire with a diameter of 150 µm diameter, was used. The tungsten microprobe tip was designed for stable measurements with a repeatable operation function, ensuring that the local contact potential difference (CPD) remained unchanged throughout the testing process. The microprobe was positioned approximately 100 μm above the surface of the conductive sample. A specific area of 2500 × 2500 μm was scanned to assess changes in the CPD distribution. The scanning was conducted in height-tracking mode, allowing the microprobe to adjust its vertical deflection within a range of 0.3 to 1.0 mm to maintain a constant distance from the sample surface. The microprobe tip and the sample surface effectively created a parallel plate capacitor configuration. This setup allowed for the measurement of the CPD, which is essentially the voltage difference that arises at the contact point between two conductors. This voltage difference provides insights into the electronic properties of the materials being tested.

The measurement data were recorded and the results were analyzed using Scanning Electrochemical Work Station M370 Version 2.45 software. The obtained surface maps of the CPD distribution were the basis for obtaining histograms of the CPD distribution with a Gaussian fitting curve. The variations in the CPD values across the surfaces of the tested materials were highly sensitive indicators of surface phenomena associated with the modifications of the NiTi alloy. This sensitivity allowed for the detection of subtle changes in the surface properties of the alloy, providing valuable insights into the effects of surface modification processes.

## 3. Results and Discussion

The Ag-TiO_2_ and Ag-TiO_2_ layers doped with hydroxyapatite were prepared by electrophoretic deposition and subsequently heat-treated at 800 °C for 2 h [33,34]. The manufacturing parameters used resulted in the creation of a new generation of composite layers with a structure significantly different from the starting materials.

The Ag-TiO_2_ coating exhibited an island-like morphology. A thin film (interlayer), comprising Ag, Ag_x_O, non-stoichiometric titanium oxide particles, and an Ag-Ti-related interphase, was formed directly on the NiTi substrate. The islands were predominantly composed of highly defective rutile. Additionally, particles with core-shell structures, featuring a carbon-layered silver core, were also identified within the layer [33].

The homogeneous HAp-Ag-TiO_2_ composite layer, with a thickness of 2 µm, contained hydroxyapatite (HAp), carbonate apatite (CHAp), metallic silver, silver oxides, Ag@C, and defective rutile [34].

The produced layers underwent thorough morphological and structural characterization [33,34], as well as an extensive evaluation of their functional and performance properties. These assessments included adhesion to the NiTi substrate, roughness, wettability, biocompatibility, antibacterial properties, and corrosion resistance.

### 3.1. Adhesion of the Coatings

Assessing mechanical properties and ensuring strong adhesion between the coating and the metallic substrate are crucial for coated implants. Strong adhesion is vital for maintaining the durability and functionality of the implant. Therefore, measurements of critical loads provided valuable insights into the scratch resistance and mechanical integrity of the deposited coatings under progressively increasing loads.

**Figure 2:**
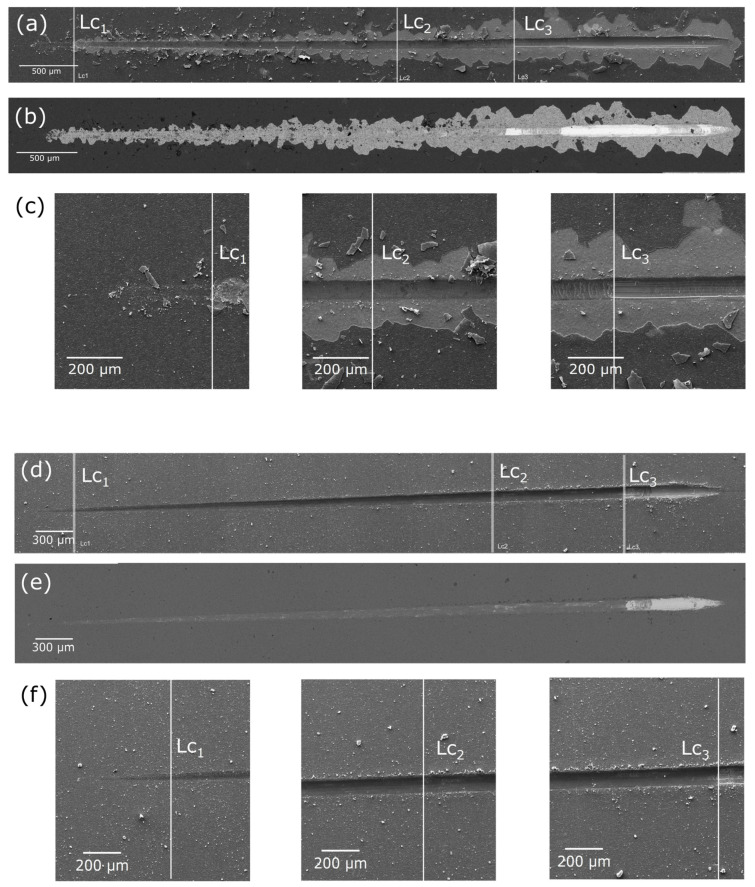
SE images (**a**,**c**,**d**,**f**) and BSE images (**b**,**e**) of Ag-TiO_2_ (**a**–**c**) and HAp-Ag-TiO_2_ (**d**–**f**) coatings after scratch test with marked critical load (Lc_1_–Lc_2_).

Both Ag-TiO_2_ coatings and those doped with hydroxyapatite exhibited very good adhesion to the substrate (Table 1). For each sample, three distinct critical load values were identified (Lc_1_, Lc_2_, and Lc_3_). Microscopic observations (Figure 2) revealed that the mechanical behavior of the individual coatings under load was completely different.

For the Ag-TiO_2_ coating, the load when the first damage occurred (Lc_1_) was characterized by a large spalling area at the interface, referred to as gross spallation. Lc_2_ is the load when the first Hertzian tensile cracks, known as arc tensile cracks, appeared within the scratch trace. Lc_3_ is the load when the layer experienced complete failure and was entirely damaged (Figure 2a).

In contrast, the hydroxyapatite-doped coating demonstrated different behaviors under load (Figure 2b). The addition of hydroxyapatite nanoparticles imparted plastic properties to the coating. In this case, the Lc_1_ is the load when the first plastic deformations of the coating were observed. Under Lc_2_, the first discontinuous plastic perforations appeared in the coating, while under Lc_3_, the layer was completely damaged, resulting in a continuous plastic perforation of the coating.

Comparative analysis of these results indicates that the hydroxyapatite-Ag-TiO_2_ coating has better adhesion and greater promise for medical applications due to its plastic deformation characteristics. This plasticity is particularly advantageous in situations involving deformations related to the shape memory effect. Conversely, the Ag-TiO_2_ coating, being more brittle, is more prone to cracking and delamination under stress. Furthermore, when compared to TiO_2_-HAp layers electrophoretically deposited on a Ti–6Al–4V alloy and heat-treated at a higher temperature (850 °C), the produced layers demonstrate significantly better adhesion. This improvement is likely due to the inclusion of reactive silver in the nanocomposite and the resultant formation of a distinctly different structure [43].

### 3.2. Topography of the Coatings

Nanoscale roughness should be optimized to support osseointegration at the cellular and molecular levels. Nanostructured surfaces can significantly enhance the interaction between the implant and bone cells, increase bone cell adhesion due to a larger contact surface, promote the differentiation of stem cells into osteoblasts, and boost the production of extracellular matrix proteins that are essential for bone formation. However, excessively rough surfaces may encourage bacterial adhesion and colonization [17,19,44].

**Figure 3:**
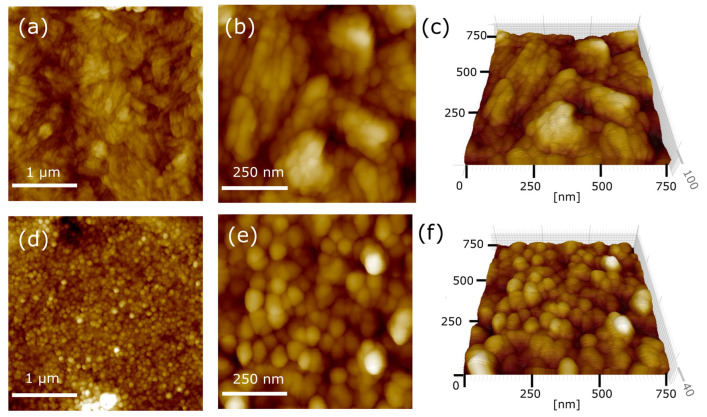
AFM topography of the Ag-TiO_2_ (**a**–**c**) and HAp-Ag-TiO_2_ (**d**–**f**) coatings.

Micro- and nanoscale morphology and microstructure examined by atomic force microscopy (AFM) revealed minute differences between the coatings. The roughness values (RMS) measured from areas of different sizes were as follows: for the Ag-TiO_2_ coating, 98 nm (10 µm × 10 µm), 39 nm (3 µm × 3 µm), and 23 nm (750 nm × 750 nm); and the hydroxyapatite-doped coating, 45 nm (10 µm × 10 µm), 20.5 nm (3 µm × 3 µm), and 15 nm (750 nm × 750 nm). HAp-Ag-TiO_2_ coatings exhibited slightly lower roughness. Moreover, an AFM investigation confirmed the microscopic observation [33,34]. The nanoparticles adhered tightly to each other, forming a compact coating (Figure 3).

### 3.3. Wettability of the Coatings

**Figure 4:**
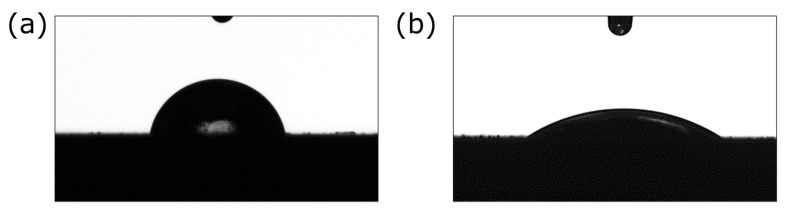
Image of a drop of water on the surface of the Ag-TiO_2_ (**a**) and the HAp-Ag-TiO_2_ coating (**b**).

Surface wettability is crucial in shaping implant properties, affecting the absorption of molecules that promote fibroblast adhesion while potentially resisting bacterial colonization at the implant–tissue interface [24,25]. The studies demonstrated that the synthesized coatings exhibit favorable hydrophilic properties (Figure 4). The contact angle measurements indicated that the Ag-TiO_2_ layer had a contact angle of 75.6 ± 2.9°, whereas the hydroxyapatite-doped layer exhibited a significantly lower contact angle of 33.7 ± 3.0°. The rougher coating displayed reduced hydrophilicity. The observed increase in hydrophilicity for the hydroxyapatite-doped coatings is attributed to the presence of hydrophilic functional groups.

### 3.4. Biocompatibility of the Coatings

**Figure 5:**
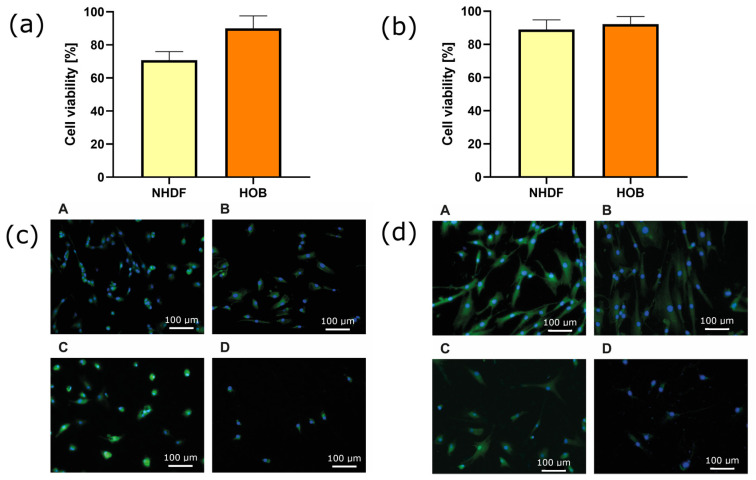
Cytotoxicity of the Ag-TiO_2_ (**a**) and HAp-Ag-TiO_2_ (**b**) against fibroblast (NHDF) and osteoblast (HOB) cells. Microscope imaging of A—NHDF cell line grown on the coatings, B—NHDF cell line grown on the NiTi substrate, C—HOB cell line grown on the coatings, and D—HOB cell line grown on the NiTi substrate for Ag-TiO_2_ (**c**) and HAp-Ag-TiO_2_ (**d**) coatings.

After performing a cytotoxicity assay for the deposited coatings by measuring mitochondrial activity (MTS assay), it is concluded that both of the tested materials are not toxic to fibroblast and osteoblast cell lines (Figure 5a,b). Cell survival rates did not decrease below 70%. The studies indicated that fibroblast survival was slightly lower on Ag-TiO_2_ layers compared to layers doped with hydroxyapatite. However, the survival rates of osteoblasts were comparable on both types of layers.

The fluorescence microscope images presented above indicate the good adhesion properties of the samples tested (Figure 5c,d). The morphology of the cells indicates their good condition, as evidenced by the formation of protrusions indicating adhesion to the surface. The cell nuclei are clearly visible and show no signs of fragmentation. The cell membrane and structure of the whole cell are compact and visible and indicate normal proliferation.

### 3.5. Antimicrobial Properties of the Coatings

Implant-associated infections typically begin with bacterial contamination during surgery. Biomaterials are used in various medical procedures and interact with different tissues throughout the body. Even minor tissue responses to implants can alter immune defenses and increase vulnerability to infection [45]. To prevent an increased risk of infection, the effect of biomaterials on the induction of microbial growth within implants becomes essential. The research presented here aimed to verify the tested materials’ impact on developing selected bacterial strains.

**Figure 6:**
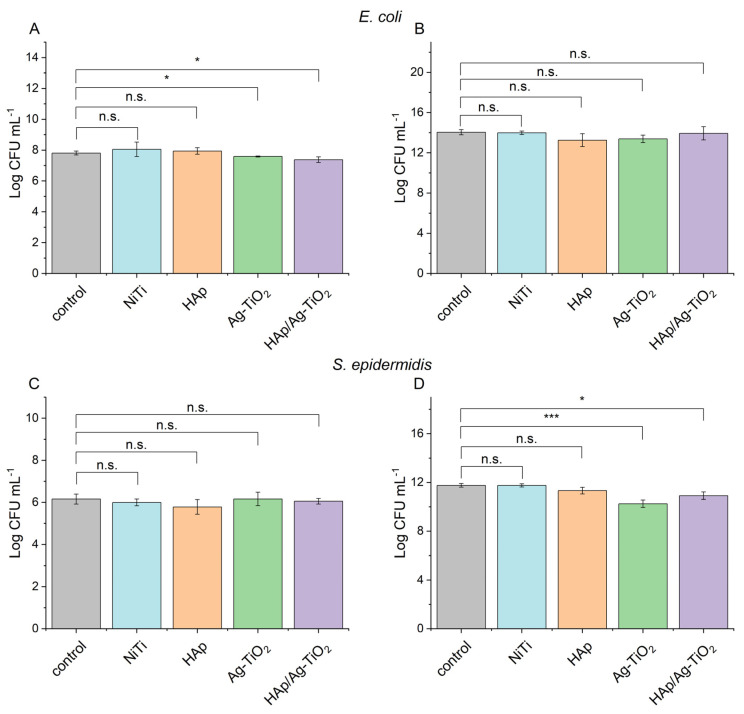
Log CFU mL^−1^ value for *E. coli* incubated in Ringer’s solution (**A**) in culture medium (**B**), and for *S. epidermidis* in Ringer’s solution (**C**) in culture medium (**D**). Mean + SD (n = 3) with a marked level of significance (* *p* < 0.05 and *** *p* < 0.005).

**Figure 7:**
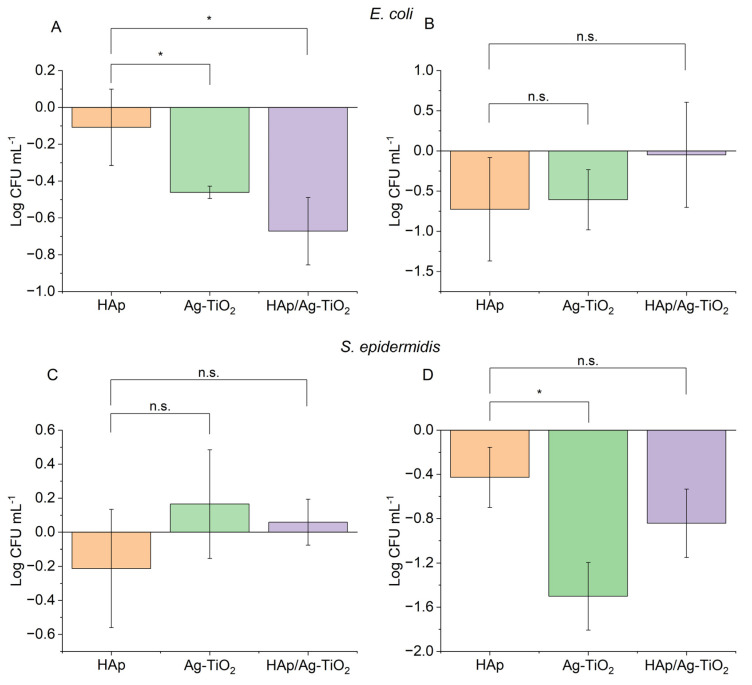
Log CFU mL^−1^ value for *E. coli* incubated in Ringer’s solution (**A**), in culture medium (**B**), and for *S. epidermidis* in Ringer’s solution (**C**), in culture medium (**D**). Mean +SD (n = 3) with a marked level of significance (* *p* < 0.05).

The tested materials showed no stimulating effect on the growth of *E. coli* and *S. epidermidis* microorganisms, both under optimal conditions for bacterial growth and under unfavorable conditions that mimic human body fluids. The results show a statistically significant inhibitory effect on the tested microorganisms’ growth, depending on the environment and bacterial strain (Figure 6). Ag-TiO_2_ coatings showed the best antibacterial properties against *S. epidermidis* when cultured in a microbial medium, while HAp-Ag-TiO_2_ coatings revealed better antibacterial properties against *E. coli* under conditions mimicking human body fluids. No statistically significant differences existed between the untreated bacteria and the NiTi substrate and HAp coatings. In a further analysis of the Ag-TiO_2_ and HAp-Ag-TiO_2_ coatings, the value obtained for the NiTi sample was used as a reference. The results are presented as the value being the difference between the HAp, Ag-TiO_2_, and HAp-Ag-TiO_2_ coatings and the NiTi substrate reference shown in Figure 7.

The value obtained for the reference (NiTi substrate) was subtracted from the value for the test materials, and the results are shown in Figure 7. For *E. coli* incubated in Ringer’s solution, a statistically significant decrease in Log CFU mL^−1^ values was observed for Ag-TiO_2_ (*p* < 0.05) and HAp-Ag-TiO_2_ (*p* < 0.05) relative to HAp (Figure 7A). An increase in the antibacterial properties of HAp-Ag-TiO_2_ against HAp for *E. coli* was observed. A study by Fu et al. also showed that adding HAp to MoS_2_-Ti6 implant coatings increases the antibacterial properties against *E. coli* and *S. aureus* compared to metallic and MoS_2_-Ti_6_ implants [46]. Under the optimal conditions provided by the microbial media, a slight decrease in CFU mL^−1^ values can also be observed. Still, it is not statistically significant (Figure 7B). The results are most likely due to the heterogeneous HAp layer. The HAp-Ag-TiO_2_ coating is characterized by HAp-rich areas, which can benefit bacterial growth and highly toxic Ag@C areas [34], which reflects the sizeable statistical deviation obtained in three independent experiments.

For *S. epidermidis*, no statistically significant differences were observed in the system with Ringer’s solution. The lack of an apparent antibacterial effect for systems carried out in Ringer’s liquid for *S. epidermidis* may be due to the sluggishness of cell metabolism due to the lack of optimal conditions for growth, resulting in no significant differences between the materials tested. However, a statistically significant decrease in Log CFU mL^−1^ values for Ag-TiO_2_ coating (*p* < 0.05) relative to HAp was observed for systems run in dedicated bacterial media (Figure 7D). Nazarov et al. showed increased antibacterial properties in samples containing a TiO_2_-Ag layer against *E. epidermidis* compared to titanium alone or with silver nanoparticles [47]. The presence of HAp in the structure of the nanomaterial as a factor that stimulates cell adhesion [48] can affect the development of a bacterial biofilm on the surfaces of implants, which increases bacterial survival. The obtained results of the study present this relationship. Both Ag-TiO_2_ and HAp-Ag-TiO_2_ have an inhibitory effect on the growth of *S. epidermidis* concerning the references and HAp. However, HAp-Ag-TiO_2_ interacts to a lesser extent than Ag-TiO_2_ concerning the tested strain in systems conducted in the microbiological medium.

### 3.6. Corrosion Resistance of the Coatings and Electronic Properties

Corrosion resistance is crucial due to the action of aggressive body fluids that cause degradation of the implant material. Current state-of-the-art corrosion testing methods, including open circuit potential testing, potentiodynamic testing, and electrochemical impedance spectroscopy, were used to study the in vitro corrosion resistance of the obtained materials.

#### 3.6.1. Open Circuit Potential Measurements

**Figure 8:**
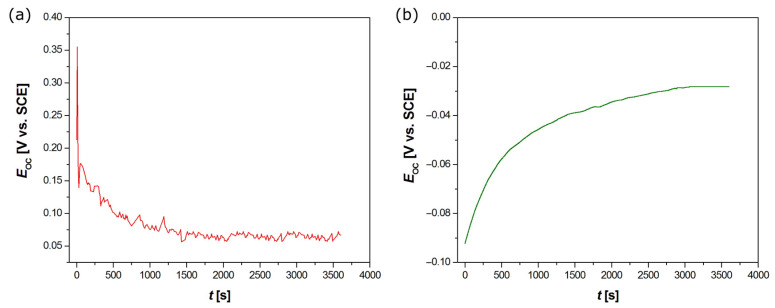
Open circuit potential (E_OC_) as a function of immersion time (t) in the Ringer’s solution at 37 °C for Ag-TiO_2_ (**a**) and HAp-Ag-TiO_2_ (**b**) electrodes.

The preliminary assessment of corrosion resistance of the Ag-TiO_2_ and HAp-Ag-TiO_2_ electrodes was performed based on the measurements of the open circuit potential, which was considered to be the approximate E_cor_. Operating at an open potentiostat loop allowed for the measurement of the signals spontaneously generated by the corroding interface of electrode–electrolyte without any perturbation caused by external polarization. The course of the E_OC_ = f(t) curve for the Ag-TiO_2_ electrode reveals that E_OC_ stabilization occurred already after approximately 1500 s (Figure 8a). The E_OC_ value decreased from 0.355 V in the first seconds to 0.067 V after 3600 s. The positive E_OC_ value obtained after 1 h of immersion in Ringer’s solution indicates the high resistance of the Ag-TiO_2_ coating to electrochemical corrosion.

It should be noted that the curve shown in Figure 8a is characterized by small fluctuations in E_OC_ during the measurement period. The E_OC_ is a measure of the voltage difference between the WE and the RE in an electrochemical cell when no current is intentionally passed through the cell. When an electrochemical reaction occurs at an electrode, it can lead to the accumulation of charge or the depletion of reactants near the electrode surface, which can cause changes in the E_OC_. Moreover, the electrode surface is surrounded by a layer of ions from the electrolyte, known as the electrical double layer. Changes in this layer, due to ion movement or adsorption/desorption processes, can also affect the E_OC_. Additionally, if there are concentration gradients in the electrolyte, the diffusion of ions to or from the electrode surface can cause fluctuations in the E_OC_. Changes in temperature can affect the E_OC_ due to changes in the electrode kinetics and the activity of ions in the electrolyte. Environmental factors, such as vibrations, electromagnetic fields, or changes in the electrolyte (e.g., pH, composition), can also cause fluctuations in the E_OC_. Some electrodes may inherently be unstable or undergo changes over time, such as corrosion or passivation, which can lead to fluctuations in the E_OC_. The measurement equipment itself can introduce noise or artifacts into the data, especially if the signal is amplified or if there are issues with the electrical connections.

In Figure 8b, the E_OC_ profile vs. t for the HAp-Ag-TiO_2_ electrode showed an increase in the potential value during the first 3000 s before a plateau appeared, which suggests an improvement of corrosion resistance with the immersion time in Ringer’s solution. The E_OC_ value increased from −0.092 V in the first seconds to −0.028 V after 3600 s. In the case of the HAp-Ag-TiO_2_ electrode, the stabilized E_OC_ value is negative, which indicates a weakening of the corrosion resistance in comparison with a Ag-TiO_2_ coating. Lower E_OC_ values compared to the NiTi electrode were also observed in the case of hydroxyapatite–silver–silica hybrid coatings [32]. The obtained results showed that the destruction processes will start earlier in the case of a coating with hydroxyapatite. The difference in the pitting resistance of the tested materials is mainly due to their chemical composition, structure, and finishing condition. The obtained results are in accordance with the well-known dependence that polished surfaces display higher resistance to pitting [3].

#### 3.6.2. Electrochemical Impedance Spectroscopy Study

**Figure 9:**
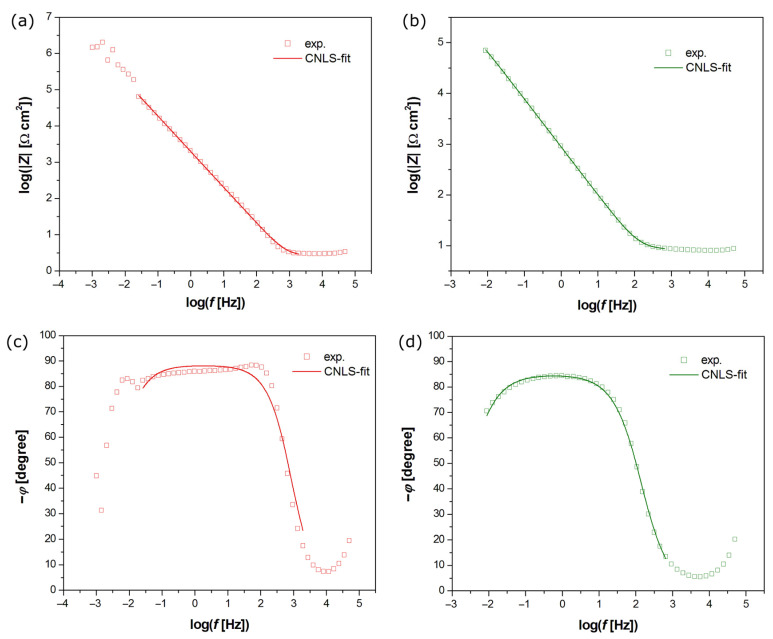
Experimental (symbols) and simulated (CNLS-fit) Bode diagrams as a function of the logarithm of the frequency. (**a**) Logarithm of the impedance modulus for Ag-TiO_2_ electrode. (**b**) Logarithm of the impedance modulus for HAp-Ag-TiO_2_ electrode. (**c**) Phase angle shift for Ag-TiO_2_ electrode; (**d**) Phase angle shift for HAp-Ag-TiO_2_ electrode.

The EIS method was used to determine the mechanism and kinetics of the electrochemical corrosion process, along with the capacitive characteristics of the tested electrodes. The experimental Bode diagrams for the coatings recorded in the Ringer’s solution at 37 °C are displayed as symbols in Figure 9. The dependence of log|Z| = log(f) in the mid-frequency range showed a slope of about −1 (Figure 9a,b). The impedance module (|Z|) was normalized by the electrode surface area and reported in Ω cm² to account for differences in electrode size. The higher value of log|Z| at f = 10 mHz equal to 5.56 Ω cm^2^ is observed for the Ag-TiO_2_ coating, as compared to the 4.85 Ω cm^2^ for the HAp-Ag-TiO_2_ coating. The parameter log|Z|_f=10mHz_ can be used for the comparative assessment of the in vitro corrosion resistance of the tested materials, which shows deterioration of the protective properties in the case of the HAp-Ag-TiO_2_ coating.

The dependence of the phase angle (*φ*) as a function of log *f* is shown in Figure 9c,d, where the same symbols are used as in Figure 9a,b. One can see a plateau in the mid-frequency range for both electrodes, which confirms the strong barrier properties of the passive layer on the electrode surface. The maximum value of *φ* is slightly less than −90°. For both types of tested electrodes, only one time constant is present in the electrical circuit. Such impedance behavior characterizes titanium and its alloys coated with a thin oxide layer in a biological milieu [49,50]. The experimental high values of |Z|_f→0_ (Figure 9a,b) and *φ* (Figure 9c,d) are typical for metallic electrodes covered with an oxide layer with capacitive behavior and high corrosion resistance [3]. 

To interpret the EIS results in terms of the protective properties of the layers on the Ni-Ti electrode substrate, the experimental data were approximated using the electrical equivalent circuit, in the form of a modified Randles circuit, shown in Figure 3. The model used for electrochemical corrosion allows for the simulation of the response of an electrical equivalent circuit and then the fitting of the circuit parameters to the experimental ESI data using the CNLS method [41]. This equivalent electrical circuit model for the pitting corrosion process displays only one semicircle on the Nyquist plot and has four adjustable parameters, including R_1_, CPE-T_1_, CPE-ϕ_1_, and R_2_ [32,41]. R_s_ is related to solution resistance in this model. R_ct_ represents the charge-transfer resistance across the interface of layer and Ringer’s solution, and CPE_dl_ is a constant phase element (CPE) introduced instead of a capacitor, which corresponds to the double-layer capacitance (C_dl_). This procedure is typically used to facilitate fitting for metallic materials covered with oxide films, whose EIS spectra deviate from the classical Randles electrical equivalent [41]. The CPE impedance is defined by Equation (1):

(1) $${\hat{Z}}_{\mathrm{CPE}}=\frac{1}{T{\left(j\omega \right)}^{\phi }}$$

where T is the capacitive parameter of CPE [F cm^−2^ s^ϕ−1^], and ϕ is the exponent of CPE related to the constant phase angle, α = 90°(1 − ϕ), which is dimensionless and takes values ≤1.

**Figure 10:**
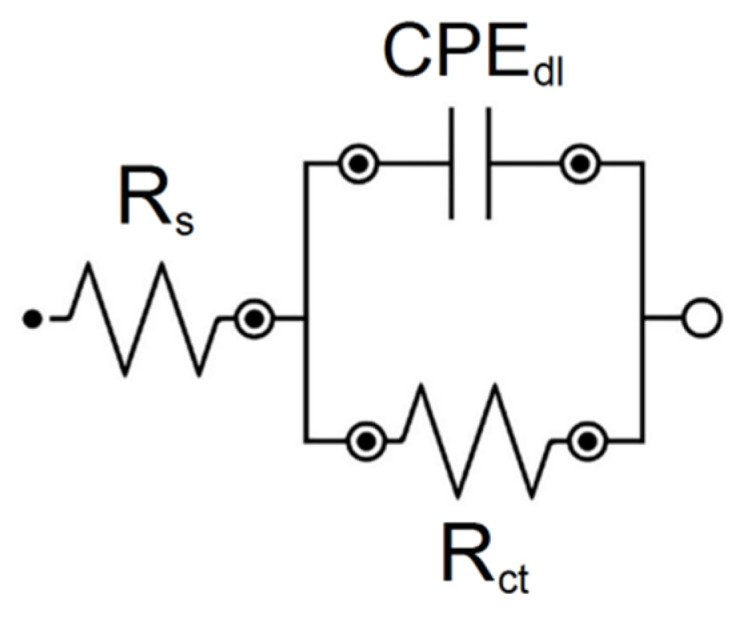
Electrical equivalent circuit for pitting corrosion used to model experimental EIS data for electrodes in Ringer’s solution at 37 °C.

Figure 9 illustrates the CNLS-fitted data marked as continuous lines that were obtained using the electrical equivalent circuits shown in Figure 10. The very good quality of the CNLS fit is visible. All CNLS-fit parameters determined using the proposed equivalent electrical circuit model for the coatings are summarized in Table 2.

The value of R_ct_ = (4.40 ± 0.01)⋅10^5^ Ω cm^2^ is determined for the Ag-TiO_2_ coating, which is ca. 1.7 times higher in comparison with the R_ct_ value for the coating with hydroxyapatite (Table 2). The physical and chemical meaning of the kinetic R_ct_ parameter relates to the ongoing corrosion process. The obtained results indicate stronger barrier properties of the surface layer directly adjacent to the NiTi substrate. In corrosion studies, a lower R_ct_ can indicate higher susceptibility to corrosion due to easier electron transfer. At the same time, a higher T_dl_ value of (8.37 ± 0.16)⋅10^−5^ F cm^−2^ s ^ϕ−1^ for the Ag-TiO_2_ electrode compared to (2.00 ± 0.01)⋅10^−4^ F cm^−2^ s ^ϕ−1^ for the HAp-Ag-TiO_2_ electrode indicates greater conductivity of the protective layer on the surface of the coating with hydroxyapatite and its electrochemical activity (Table 2). The deviation of the CPE-ϕ_1_ parameter from one can be related to physico-chemical or geometrical inhomogeneities [41].

#### 3.6.3. Susceptibility to Pitting Corrosion

**Figure 11:**
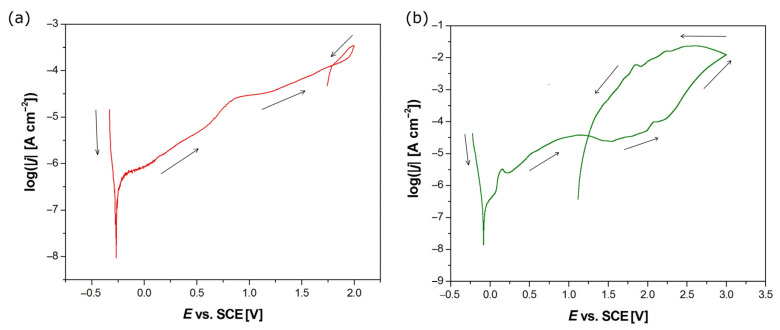
Cyclic potentiodynamic polarization curve at the polarization scan rate of v = 1 mV s^−1^ for the electrode in the Ringer’s solution at 37 °C for Ag-TiO_2_ (**a**) and HAp-Ag-TiO_2_ (**b**) coatings. The arrows indicate the polarization scan direction.

The susceptibility to pitting corrosion of the tested electrodes in the Ringer’s solution at 37° was determined based on cyclic potentiodynamic polarization curves presented in a semi-logarithmic scale (Figure 11). The obtained log|j| = f(E) dependences were the basis for the determination of the key parameters of corrosion resistance, such as E_cor_, E_bd_, and E_p_ (Table 3).

The analysis of the obtained cyclic potentiodynamic polarization curves revealed the passive behavior of both types of tested electrodes. A minimum shift on the log|j| = f(E) curve towards anodic potentials is visible for the HAp-Ag-TiO_2_ electrode, which suggests higher corrosion resistance in comparison with the Ag-TiO_2_ electrode. However, the curve shift to more anodic potentials is accompanied by an increase in current density, which cannot be interpreted as an indication of increased corrosion resistance for the HAp-Ag-TiO_2_ electrode. The E_cor_ is a valuable comparative parameter for assessing the corrosion resistance of materials (Table 4). It provides a basis for ranking materials, evaluating the impact of environmental factors, and designing corrosion protection strategies. However, it is important to consider the dynamic nature of E_cor_ and other factors when interpreting these values. For example, the electrode’s surface may be less reactive due to changes in surface chemistry or microstructure, leading to a higher onset potential for significant oxidation, or corrosion inhibitors can adsorb on the electrode surface and block active sites, shifting the curve to more anodic potentials. A shift towards more anodic potentials may also indicate an increase in the pitting potential (Table 4). The E_cor_ is the potential at which the metal is in equilibrium with its environment, meaning the rates of anodic (oxidation) and cathodic (reduction) reactions are equal. In the range of potentials with more cathodic values than the E_cor_, both investigated electrodes are corrosion-resistant (Figure 11). After exceeding the E_cor_ value on the anodic potentiodynamic curve, the oxidation process begins at more positive potentials, which can result in electrode dissolution, passivation, or pitting. This means that the rate of the anodic (oxidation) reaction becomes greater than the rate of the cathodic (reduction) reaction.

The observed passive current densities in Figure 11 are typical for titanium and its alloys in the biological environment [32]. The passive range ends with a potential of about 1.986 V for the Ag-TiO_2_ electrode, while in the case of the HAp-Ag-TiO_2_, the destruction of the protective layer occurs later at the E_bd_ of about 2.590 V. It should be noted that the E_bd_ is dependent on the polarization scan rate, as anodic dissolution is a kinetically controlled process [42]. Pitting may be initiated by a slight surface defect, such as a scratch, local change in composition, or damage to the protective coating. For potentials above the E_bd_, an increase in the current density is observed with the increase in the anodic potential due to the oxidation of metal cations forming the passive layers. The obtained return curves in Figure 11a,b do not coincide with the primary curves at some distance. The point of intersection of both curves corresponds to E_p_. The E_bd_ is located near the breaking point of the anodic polarization curve. Pitting initiation can only occur at potentials more positive than E_bd_. Meanwhile, at potentials more negative than E_p_, pitting corrosion does not occur, and existing pits are repassivated. In the potential range from E_p_ to E_bd_, new pits do not form, but existing pits can develop. The width of the hysteresis loop formed by the polarization curve indicates the greater susceptibility of the HAp-Ag-TiO_2_ coating to pitting corrosion.

#### 3.6.4. Electronic Properties

**Figure 12:**
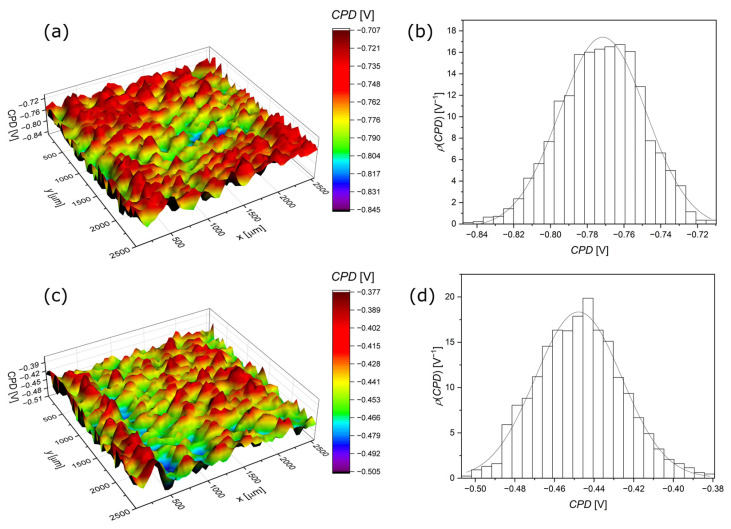
CPD surface distribution map (**a**,**c**) and CPD histogram with Gaussian fit curve (**b**,**d**) for Ag-TiO_2_ (**a**,**b**) and HAp-Ag-TiO_2_ (**c**,**d**) coatings.

Figure 12a,c shows the CPD surface distribution maps for Ag-TiO_2_ and HAp-Ag-TiO_2_, respectively. On the basis of the CPD surface distribution maps, where CPD is the variable z, the histograms of the CPD distribution were obtained, as shown in Figure 12b,d.

Table 4 presents the values of the statistical parameters as the arithmetic average of CPD heights (CPD_av_), the root-mean-square deviation of CPD heights (CPD_rms_), the skewness (CPD_sk_), and the excess kurtosis (CPD_ku_), which were determined based on the CPD surface distribution maps in Figure 12a,c. These parameters characterize the surface condition of the tested coatings.

One can see that CPD_av_ is about 1.7 times lower for the Ag-TiO_2_ coating compared to the HAp-Ag-TiO_2_ (Table 4). The increase in CPD_av_ in the case of the HAp-Ag-TiO_2_ indicates a smaller electrochemically active surface, which may be related to lower porosity and/or surface roughness compared to the Ag-TiO_2_ coating, which confirmed the AFM measurements (Figure 3). CPD_q_ is slightly lower for the coating with hydroxyapatite and equals ca. 21.5 mV (Table 4). A CPD_q_ value of ca. 21.5 mV is slightly lower for the HAp-Ag-TiO_2_ and indicates a more uniform surface (Table 4). The shape of the CPD distribution is quantitatively described by CPD_sk_ and CPD_ku_, whose values indicate that the CPD distribution is of a Gaussian type. CPD_sk_ and CPD_ku_ values close to zero testify that the CPD heights are distributed symmetrically around the average, and there are no areas with relatively large/small CPD values on the surfaces of both coatings.

## 4. Conclusions

To functionalize the NiTi alloy, innovative multifunctional nanolayers composed of Ag-TiO_2_ and Ag-TiO_2_ composites doped with hydroxyapatite were synthesized on its surface. These coatings were characterized with respect to surface topography and crucial functional properties, including adhesion, surface wettability, biocompatibility, antibacterial efficacy, and corrosion resistance. Furthermore, the electrochemical corrosion kinetics and mechanisms were investigated to elucidate their protective performance.

The hydroxyapatite coatings demonstrated superior adhesion to the NiTi substrate and enhanced plasticity, making them particularly promising for medical applications. These coatings also exhibited a lower surface roughness and increased hydrophilicity, which are advantageous for biological interactions.

Both types of coatings were biocompatible, supporting the adhesion and proliferation of both fibroblast and osteoblast cells on their surfaces. Importantly, neither coating promoted the growth of *E. coli* and *S. epidermidis* under optimal bacterial growth conditions or in environments simulating human body fluids. The Ag-TiO_2_ coatings exhibited the most effective antibacterial activity against *S. epidermidis* in standard microbiological environments, while the HAp-Ag-TiO_2_ coatings were more effective against *E. coli* under conditions mimicking human body fluids.

Open circuit potential measurements showed high resistance of Ag-TiO_2_ coating to electrochemical corrosion, better than for the hydroxyapatite layer. Obtained DC and AC results showed that the Ag-TiO_2_ coating is characterized by stronger barrier properties of the surface layer directly adjacent to the NiTi substrate, while the HAp-Ag-TiO_2_ coating has a higher electrochemical activity and a higher susceptibility to pitting corrosion in the Ringer’s solution. In an air environment, the HAp-Ag-TiO_2_ coating showed an increased CPD_av_ due to a lower surface roughness.

## Figures and Tables

**Table 1:** Critical load for tested coatings, where F_t_ is a friction force and AE is an acoustic emission.

	Lc_1_		Lc_2_		Lc_3_	
	F_t_ [N]	AE [%]	F_t_ [N]	AE [%]	F_t_ [N]	AE [%]
Ag-TiO_2_	1.02 ± 0.29	1.01 ± 0.28	13.42 ± 1.14	13.45 ± 1.12	20.04 ± 1.31	20.09 ± 1.30
HAp-Ag-TiO_2_	2.32 ± 0.27	2.32 ± 0.29	18.07 ± 2.09	18.08 ± 2.1	24.41 ± 3.71	24.43 ± 3.70

**Table 2:** The parameters with standard deviations determined by approximation of the experimental EIS data for the coatings in the Ringer’s solution at 37 °C (Figure 9) and the equivalent electrical circuit model for the pitting corrosion process (Figure 10).

Electrode Type	R_s_ [Ω cm^2^]	T_dl_ [F cm^−2^ s ^ϕ−1^]	ϕ_dl_	R_ct_ [Ω cm^2^]
Ag-TiO_2_	2.66 ± 0.11	(8.37 ± 0.16)⋅10^−5^	0.982 ± 0.004	(4.40 ± 0.01)⋅10^5^
HAp-Ag-TiO_2_	8.38 ± 0.06	(2.00 ± 0.01)⋅10^−4^	0.945 ± 0.001	(2.58 ± 0.09)⋅10^5^

**Table 3:** Parameters of the corrosion resistance of the coatings in the Ringer’s solution at 37 °C determined based on the cyclic potentiodynamic polarization curves (Figure 11), where E_cor_—corrosion potential, E_bd_—breakdown potential, and E_p_—protection potential.

Type of Electrode	E_cor_ [V]	E_bd_ [V]	E_p_ [V]
Ag-TiO_2_	−0.267	1.986	1.790
HAp-Ag-TiO_2_	−0.083	2.590	1.249

**Table 4:** Statistical parameters calculated using 3D maps of CPD surface distribution for the Ag-TiO_2_ and HAp-Ag-TiO_2_ samples. CPD_av_—arithmetic average, CPD_q_—root mean square, CPD_sk_—skewness, and CPD_ku_—excess kurtosis.

Type of Electrode	CPD_av_ [V]	CPD_q_ [mV]	CPD_sk_	CPD_ku_
Ag-TiO_2_	−0.770	22.2	−0.14	−0.24
HAp-Ag-TiO_2_	−0.444	21.5	0.19	−0.13

## Data Availability

The data presented in this study are openly available in RepOD https://repod.icm.edu.pl/dataset.xhtml?persistentId=doi:10.18150/64KWUO (accessed on 13 August 2024).

## References

[1] Yoneyama T., Miyazaki S. (2008). Shape Memory Alloys for Biomedical Applications.

[2] Lelątko J., Goryczka T. (2013). Modyfikacja Powierzchni Stopów NiTi Wykazujących Pamięć Kształtu.

[3] Smołka A., Dercz G., Rodak K., Łosiewicz B. (2015). Evaluation of Corrosion Resistance of Nanotubular Oxide Layers on the Ti13Zr13Nb Alloy in Physiological Saline Solution. Arch. Metall. Mater..

[4] Dudek K., Dulski M., Goryczka T., Gerle A. (2018). Structural Changes of Hydroxyapatite Coating Electrophoretically Deposited on NiTi Shape Memory Alloy. Ceram. Int..

[5] Vdoviaková K., Jenca A., Danko J., Kresáková L., Simaiová V., Reichel P., Rusnák P., Pribula J., Vrzgula M., Askin S.J. (2023). Regenerative Potential of Hydroxyapatite-Based Ceramic Biomaterial on Mandibular Cortical Bone: An In Vivo Study. Biomedicines.

[6] Dorozhkin S.V. (2017). Hydroxyapatite & Other Calcium Orthophosphates: Nanodimensional, Multiphasic & Amorphous Formulations.

[7] Yazdani J., Ahmadian E., Sharifi S., Shahi S., Maleki Dizaj S. (2018). A Short View on Nanohydroxyapatite as Coating of Dental Implants. Biomed. Pharmacother..

[8] Lama-Odría M.d.C.D., del Valle L.J., Puiggalí J. (2022). Hydroxyapatite Biobased Materials for Treatment and Diagnosis of Cancer. Int. J. Mol. Sci..

[9] Lara-Ochoa S., Ortega-Lara W., Guerrero-Beltrán C.E. (2021). Hydroxyapatite Nanoparticles in Drug Delivery: Physicochemistry and Applications. Pharmaceutics.

[10] Wu B., Tang Y., Wang K., Zhou X., Xiang L. (2022). Nanostructured Titanium Implant Surface Facilitating Osseointegration from Protein Adsorption to Osteogenesis: The Example of TiO2 NTAs. Int. J. Nanomed..

[11] Verma R., Gangwar J., Srivastava A.K. (2017). Multiphase TiO_2_ Nanostructures: A Review of Efficient Synthesis, Growth Mechanism, Probing Capabilities, and Applications in Bio-Safety and Health. RSC Adv..

[12] Akshaya S., Rowlo P.K., Dukle A., Nathanael A.J. (2022). Antibacterial Coatings for Titanium Implants: Recent Trends and Future Perspectives. Antibiotics.

[13] Ali A., Polepalli L., Chowdhury S., Carr M.A., Janorkar A.V., Marquart M.E., Griggs J.A., Bumgardner J.D., Roach M.D. (2024). Silver-Doped Titanium Oxide Layers for Improved Photocatalytic Activity and Antibacterial Properties of Titanium Implants. J. Funct. Biomater..

[14] Chouirfa H., Bouloussa H., Migonney V., Falentin-Daudré C. (2019). Review of Titanium Surface Modification Techniques and Coatings for Antibacterial Applications. Acta Biomater..

[15] Lin L., Wang H., Ni M., Rui Y., Cheng T.-Y., Cheng C.-K., Pan X., Li G., Lin C. (2014). Enhanced Osteointegration of Medical Titanium Implant with Surface Modifications in Micro/Nanoscale Structures. J. Orthop. Transl..

[16] Wong M., Eulenberger J., Schenk R., Hunziker E. (1995). Effect of Surface Topology on the Osseointegration of Implant Materials in Trabecular Bone. J. Biomed. Mater. Res..

[17] Gittens I.R.A., McLachlan T., Cai Y., Berner S., Tannenbaum R., Schwartz Z., Sandhage K.H., Boyan B.D. (2011). The Effects of Combined Micron-/Submicron-Scale Surface Roughness and Nanoscale Features on Cell Proliferation and Differentiation. Biomaterials.

[18] Jassim R.K. (2017). The Effect of Implant Screw Coating with Nano-Hydroxyapatite and Magnesium Chloride Mixture on Osseointegration: Biomechanical and Histological Study. Int. J. Med. Res. Health Sci..

[19] Sachin P.G., Uppoor A.S., Nayak S.U. (2022). Nano-Scale Surface Modification of Dental Implants—An Emerging Boon for Osseointegration and Biofilm Control. Acta Marisiensis—Ser. Medica.

[20] Biggs M.J.P., Richards R.G., Gadegaard N., McMurray R.J., Affrossman S., Wilkinson C.D.W., Oreffo R.O.C., Dalby M.J. (2009). Interactions with Nanoscale Topography: Adhesion Quantification and Signal Transduction in Cells of Osteogenic and Multipotent Lineage. J. Biomed. Mater. Res. A.

[21] Dalby M.J., McCloy D., Robertson M., Wilkinson C.D.W., Oreffo R.O.C. (2006). Osteoprogenitor Response to Defined Topographies with Nanoscale Depths. Biomaterials.

[22] Webster T.J., Ergun C., Doremus R.H., Siegel R.W., Bizios R. (2000). Enhanced Functions of Osteoblasts on Nanophase Ceramics. Biomaterials.

[23] Webster T.J., Ergun C., Doremus R.H., Siegel R.W., Bizios R. (2000). Specific Proteins Mediate Enhanced Osteoblast Adhesion on Nanophase Ceramics. J. Biomed. Mater. Res..

[24] Gittens R.A., Scheideler L., Rupp F., Hyzy S.L., Geis-Gerstorfer J., Schwartz Z., Boyan B.D. (2014). A Review on the Wettability of Dental Implant Surfaces II: Biological and Clinical Aspects. Acta Biomater..

[25] Rupp F., Gittens R.A., Scheideler L., Marmur A., Boyan B.D., Schwartz Z., Geis-Gerstorfer J. (2014). A Review on the Wettability of Dental Implant Surfaces I: Theoretical and Experimental Aspects. Acta Biomater..

[26] Wilson C.J., Clegg R.E., Leavesley D.I., Pearcy M.J. (2005). Mediation of Biomaterial-Cell Interactions by Adsorbed Proteins: A Review. Tissue Eng..

[27] On the Influence of Flow Conditions and Wettability on Blood Material Interactions—PubMed. https://pubmed.ncbi.nlm.nih.gov/14530069/.

[28] Implantation of Hydrophilic and Hydrophobic Titanium Discs in Rat Tibia: Cellular Reactions on the Surfaces during the First 3 Weeks in Bone—PubMed. https://pubmed.ncbi.nlm.nih.gov/15120522/.

[29] van Loosdrecht M.C., Lyklema J., Norde W., Schraa G., Zehnder A.J. (1987). The Role of Bacterial Cell Wall Hydrophobicity in Adhesion. Appl. Environ. Microbiol..

[30] Doyle R.J. (2000). Contribution of the Hydrophobic Effect to Microbial Infection. Microbes Infect..

[31] Aplin A.E., Howe A.K., Juliano R.L. (1999). Cell Adhesion Molecules, Signal Transduction and Cell Growth. Curr. Opin. Cell Biol..

[32] Dudek K., Dulski M., Łosiewicz B. (2020). Functionalization of the NiTi Shape Memory Alloy Surface by HAp/SiO_2_/Ag Hybrid Coatings Formed on SiO_2_-TiO_2_ Glass Interlayer. Materials.

[33] Optimization of the Electrophoretic Deposition Parameters and Mechanism of Formation of Ag-TiO2 Nanocoatings on a NiTi Shape Memory Alloy: Part I. https://www.mdpi.com/2079-6412/14/1/44.

[34] Dudek K., Dulski M., Podwórny J., Kujawa M., Gerle A., Rawicka P. (2024). Functionalization of the NiTi Shape Memory Alloy Surface through Innovative Hydroxyapatite/Ag-TiO2 Hybrid Coatings. Materials.

[35] (2008). Determination of Scratch Properties.

[36] (2005). Fine Ceramics (Advanced Ceramics, Advanced Technical Ceramics)—Determination of Adhesion of Ceramic Coatings by Scratch Testing.

[37] (2022). Standard Test Method for Adhesion Strength and Mechanical Failure Modes of Ceramic Coatings by Quantitative Single Point Scratch Testing.

[38] (2020). Standard Test Method for Evaluation of Scratch Resistance of Polymeric Coatings and Plastics Using an Instrumented Scratch Machine.

[39] Woźniak A., Smok W., Szewczenko J., Staszuk M., Chladek G. (2024). Influence of Hybrid Surface Modification on Biocompatibility and Physicochemical Properties of Ti-6Al-4V ELI Titanium. J. Funct. Biomater..

[40] (2021). Dentistry - Corrosion Test Methods for Metallic Materials.

[41] (2001). User Manual for Frequency Response Analysis (FRA) for Windows Version 4.9.

[42] AUTOLAB (1998). Electrochemical Instruments. Description of the Instrument.

[43] Farnoush H., Aghazadeh Mohandesi J., Çimenoğlu H. (2015). Micro-Scratch and Corrosion Behavior of Functionally Graded HA-TiO_2_ Nanostructured Composite Coatings Fabricated by Electrophoretic Deposition. J. Mech. Behav. Biomed. Mater..

[44] Paital S.R., Dahotre N.B. (2009). Calcium Phosphate Coatings for Bio-Implant Applications: Materials, Performance Factors, and Methodologies. Mater. Sci. Eng. R Rep..

[45] Shahid A., Aslam B., Muzammil S., Aslam N., Shahid M., Almatroudi A., Allemailem K.S., Saqalein M., Nisar M.A., Rasool M.H. (2021). The Prospects of Antimicrobial Coated Medical Implants. J. Appl. Biomater. Funct. Mater..

[46] Fu J., Zhu W., Liu X., Liang C., Zheng Y., Li Z., Liang Y., Zheng D., Zhu S., Cui Z. (2021). Self-Activating Anti-Infection Implant. Nat. Commun..

[47] Nazarov D., Ezhov I., Yudintceva N., Shevtsov M., Rudakova A., Kalganov V., Tolmachev V., Zharova Y., Lutakov O., Kraeva L. (2022). Antibacterial and Osteogenic Properties of Ag Nanoparticles and Ag/TiO2 Nanostructures Prepared by Atomic Layer Deposition. J. Funct. Biomater..

[48] Athukorala S.S., Liyanage C.J., Jayasundera A.C.A. (2022). Hydroxyapatite Incorporated Bacterial Cellulose Hydrogels as a Cost-Effective 3D Cell Culture Platform. Soft Mater..

[49] Szklarska M., Dercz G., Rak J., Łosiewicz B., Simka W. (2015). The Influence of Passivation Type on Corrosion Resistance of Ti15Mo Alloy in Simulated Body Fluids. Arch. Metall. Mater..

[50] Smołka A., Rodak K., Dercz G., Dudek K., Łosiewicz B. (2014). Electrochemical Formation of Self-Organized Nanotubular Oxide Layers on Ti13Zr13Nb Alloy for Biomedical Applications. Acta Phys. Pol. A.

